# Delineation of dual molecular diagnosis in patients with skeletal deformity

**DOI:** 10.1186/s13023-022-02293-x

**Published:** 2022-03-28

**Authors:** Lian Liu, Liying Sun, Yujun Chen, Muchuan Wang, Chenxi Yu, Yingzhao Huang, Sen Zhao, Huakang Du, Shaoke Chen, Xin Fan, Wen Tian, Zhihong Wu, Deciphering Disorders Involving Scoliosis and COmorbidities (DISCO) study group, Guixing Qiu, Terry Jianguo Zhang, Nan Wu

**Affiliations:** 1 Department of Orthopedic Surgery, State Key Laboratory of Complex Severe and Rare Diseases, Peking Union Medical College Hospital, Peking Union Medical College and Chinese Academy of Medical Sciences, No. 1 Shuaifuyuan, Beijing, 100730 China; 2 Graduate School of Peking Union Medical College, Beijing, 100730 China; 3 Beijing Key Laboratory for Genetic Research of Skeletal Deformity, Beijing, 100730 China; 4 Key Laboratory of Big Data for Spinal Deformities, Chinese Academy of Medical Sciences, Beijing, 100730 China; 5 Department of Hand Surgery, Beijing Jishuitan Hospital, Beijing, 100035 China; 6 The Second Affiliated Hospital of Guangxi Medical University, Nanning, 530000 Guangxi China; 7 Department of Joint Surgery, Shandong Provincial Hospital Affiliated To Shandong First Medical University, Shandong, 250021 China

**Keywords:** Skeletal deformity, Phenotypic characteristics, Dual molecular diagnosis, Medical genetics

## Abstract

**Background:**

Skeletal deformity is characterized by an abnormal anatomical structure of bone and cartilage. In our previous studies, we have found that a substantial proportion of patients with skeletal deformity could be explained by monogenic disorders. More recently, complex phenotypes caused by more than one genetic defect (i.e., dual molecular diagnosis) have also been reported in skeletal deformities and may complicate the diagnostic odyssey of patients. In this study, we report the molecular and phenotypic characteristics of patients with dual molecular diagnosis and variable skeletal deformities.

**Results:**

From 1108 patients who underwent exome sequencing, we identified eight probands with dual molecular diagnosis and variable skeletal deformities. All eight patients had dual diagnosis consisting of two autosomal dominant diseases. A total of 16 variants in 12 genes were identified, 5 of which were of de novo origin. Patients with dual molecular diagnosis presented blended phenotypes of two genetic diseases. Mendelian disorders occurred more than once include Osteogenesis Imperfecta Type I (*COL1A1*, MIM:166200), Neurofibromatosis, Type I (*NF1*, MIM:162200) and Marfan Syndrome (*FBN1*, MIM:154700).

**Conclusions:**

This study demonstrated the complicated skeletal phenotypes associated with dual molecular diagnosis. Exome sequencing represents a powerful tool to detect such complex conditions.

**Supplementary Information:**

The online version contains supplementary material available at 10.1186/s13023-022-02293-x.

## Background

Skeletal deformity is characterized by an abnormal anatomical structure of the bone and cartilage [1]. Genetic factors are essential for the pathogenesis of skeletal deformities [2]. The tenth version of the Nosology and Classification of Genetic Skeletal Disorders included 461 different diseases which can be classified into 42 groups based on their clinical, radiographic, and/or molecular findings [3]. In previous studies, we found that a substantial proportion of cases with early-onset scoliosis could be explained by monogenic disorders such as achondroplasia (MIM: 100800), Freeman–Sheldon syndrome (FSS) (MIM:193700), and spondyloepimetaphyseal dysplasia (MIM: 602557) [4–6]. In addition to monogenic conditions, complex phenotypes caused by two genetic disorders (i.e., dual molecular diagnosis) have also been reported in skeletal deformities. For example, a fetus with complex joint dislocations and congenital scoliosis was identified to be double heterozygote for putatively pathogenic *FBN1* and *FBN2* variants [7]. Tang et al. identified a patient with pathogenic variants in both *FBN1* and *PTPN11*, which caused blended phenotypes of Marfan syndrome (MIM:154700) and LEOPARD syndrome (MIM:151100) [8].

The co-existence of two Mendelian conditions challenges the diagnosis and clinical management in patients with skeletal deformities. The precise diagnosis of such conditions needs comprehensive genetic testing tools such as exome sequencing (ES) [9–11]. Thus far, systematic investigations of dual molecular diagnosis have been performed in neurodevelopmental disorders [12, 13], genetic muscle diseases [14], and endocrine dysfunction [13]. However, the phenotypic characteristics of dual diagnosis in patients with skeletal deformities are still less studied.

Here, we report eight cases with dual molecular diagnosis from the Deciphering disorders Involving Scoliosis and COmorbidities (DISCO) study. We describe the phenotypic characteristics of these patients and clinical relevance for the molecular diagnoses.

## Results

### Patients with dual molecular diagnosis and a variety of skeletal deformities

From 1108 patients who underwent exome sequencing in the DISCO study, we identified eight probands with dual molecular diagnosis and a variety of skeletal deformities (Table 1). All eight probands have dual diagnosis of two autosomal dominant (AD) diseases. A total of 16 pathogenic variants in 12 genes were identified, 5 of which were de novo. The frequently observed molecular diagnoses (observed in more than one patient) include Osteogenesis Imperfecta Type I (*COL1A1*, MIM:166200), Neurofibromatosis, Type I (*NF1*, MIM:162200) and Marfan Syndrome (*FBN1*, MIM:154700).

**Table 1:** Summary of the clinical and molecular findings of studied subjects

Case number	Case ID	Age	Sex	Inheritance	Clinical diagnosis	Gene	Molecular diagnosis	Zygosity
Case 1	SCO2003P1972	7	M	AD	CS II	*POGZ*	White–Sutton syndrome	Het
						*FBN1*	Marfan syndrome	Het
Case 2	SCO1908P0067	18	M	AD	AIS	*COL1A1*	Osteogenesis imperfecta	Het
						*FBN1*	Marfan syndrome	Het
Case 3	PCT2007P0019	8	F	AD	NFS	*NF1*	Neurofibromatosis, type 1	Het
						*COL1A1*	Osteogenesis imperfecta	Het
Case 4	SSS2008P0037	6	M	AD	GHD	*ANKRD11*	KBG syndrome	Het
						*COL11A1*	Marshall syndrome	Het
Case 5	SSS1910P0094	8	F	AD	ISS	*NF1*	Neurofibromatosis, Type 1	Het
						*GLI2*	Culler–Jones syndrome	Het
Case 6	SSS2010P0110	12	F	AD	ISS	*TP63*	Rapp-Hodgkin syndrome	Het
						*PTPN11*	Noonan syndrome	Het
Case 7	RDD2001P0005	2	M	AD	Arthrogryposis	*FBN2*	Beals syndrome	Het
						*ANKRD11*	KBG syndrome	Het
Case 8	P19009402	4	M	AD	Syndactyly	*FGFR2*	Apert syndrome	Het
						*RYR1*	Malignant hyperthermia susceptibility 1	Het

Case number	Transcript numbers	Origin	Variant type	Variant	GnomAD frequency	Gerp++	CADD	Patient phenotype
Case 1	NM_015100.3	De novo	Frameshift	c.1180_1181del p.(Met394ValfsTer9)	0	3.36	NA	Ocular hypertelorism; intellectual disability; scoliosis; congenital dislocation of hip joint
	NM_000138.4	Maternal	Nonsense	c.2649G > A p.(Trp883Ter)	0	5.33	38	
Case 2	NM_000088.3	De novo	Nonsense	c.1081C > T p.(Arg361Ter)	0	3.97	37	Bone fragility; scoliosis; osteopenia
	NM_000138.4	Maternal	Missense	c.1453C > T p.(Arg485Cys)	0	5.33	32	
Case 3	NM_000267.3	Paternal	Frameshift	c.2307del p.(Thr770LeufsTer21)	0	5.25	18.24	Cafe´-au-lait macules; scoliosis; bone fragility; blue sclera
	NM_000088.3	Paternal	Splicing	c.2028+ 4 A > G	0	NA	NA	
Case 4	NM_013275.5	De novo	Nonsense	c.4750G > T p.(Glu1584Ter)	0	5.08	48	Short stature; depressed nasal bridge; long philtrum; low-set ears; tongue thrusting
	NM_001854.3	Paternal	Frameshift	c.2508dup p.(Leu837ThrfsTer81)	0	NA	NA	
Case 5	NM_000267.3	NA	Splicing	c.6705–1 G > A	0	5.59	18	Scoliosis; short stature; abnormality of the cerebral white matter; cafe-au-Lait macules
	NM_005270.4	NA	Frameshift	c.1189del p.(Val397CysfsTer124)	0	NA	NA	
Case 6	NM_003722.4	NA	Nonsense	c.109C > T p.(Arg37Ter)	0	3.84	22.7	Short stature; low set ear; low posterior hairline; scoliosis; hyperpigmentation; webbed neck
	NM_002834.3	NA	Missense	c.1510A > G p.(Met504Val)	0	5.13	25.6	
Case 7	NM_001999.3	Paternal	Missense	c.3437A > G p.(Tyr1146Cys)	0	5.13	18.69	Joint contractures; atrial septal defect; clinodactyly of fingers
	NM_013275.5	De novo	Frameshift	c.3024_3025del p.(Lys1009GlyfsTer8)	0	NA	NA	
Case 8	NM_000141.4	De novo	Missense	c.755C > G p. (Ser252Trp)	0	5.79	23.5	Cloverleaf skull; orbital hypertelorism; proptosis; midfacial hypoplasia; syndactyly of the hands and feet; malignant hyperthermia
	NM_000540.2	Paternal	Frameshift	c.12788_12793dup p.(Glu4263_Gly4264dup)	3.36 × 10^–5^	NA	NA	

*AD* autosomal dominant, *Het* heterozygous, *NA* not applicable/not available, *CS* congenital scoliosis, *AIS* adolescent idiopathic scoliosis, *NFS* neurofibromatosis, *GHD* growth hormone deficiency, *ISS* idiopathic short stature

### The complex clinical features of patients with dual molecular diagnosis

Patients with dual molecular diagnosis presented blended phenotypes of two genetic diseases, which significantly complicated their diagnostic odysseys. Here we report the detailed clinical characteristics of these patients.

#### Case 1

**Fig. 1:**
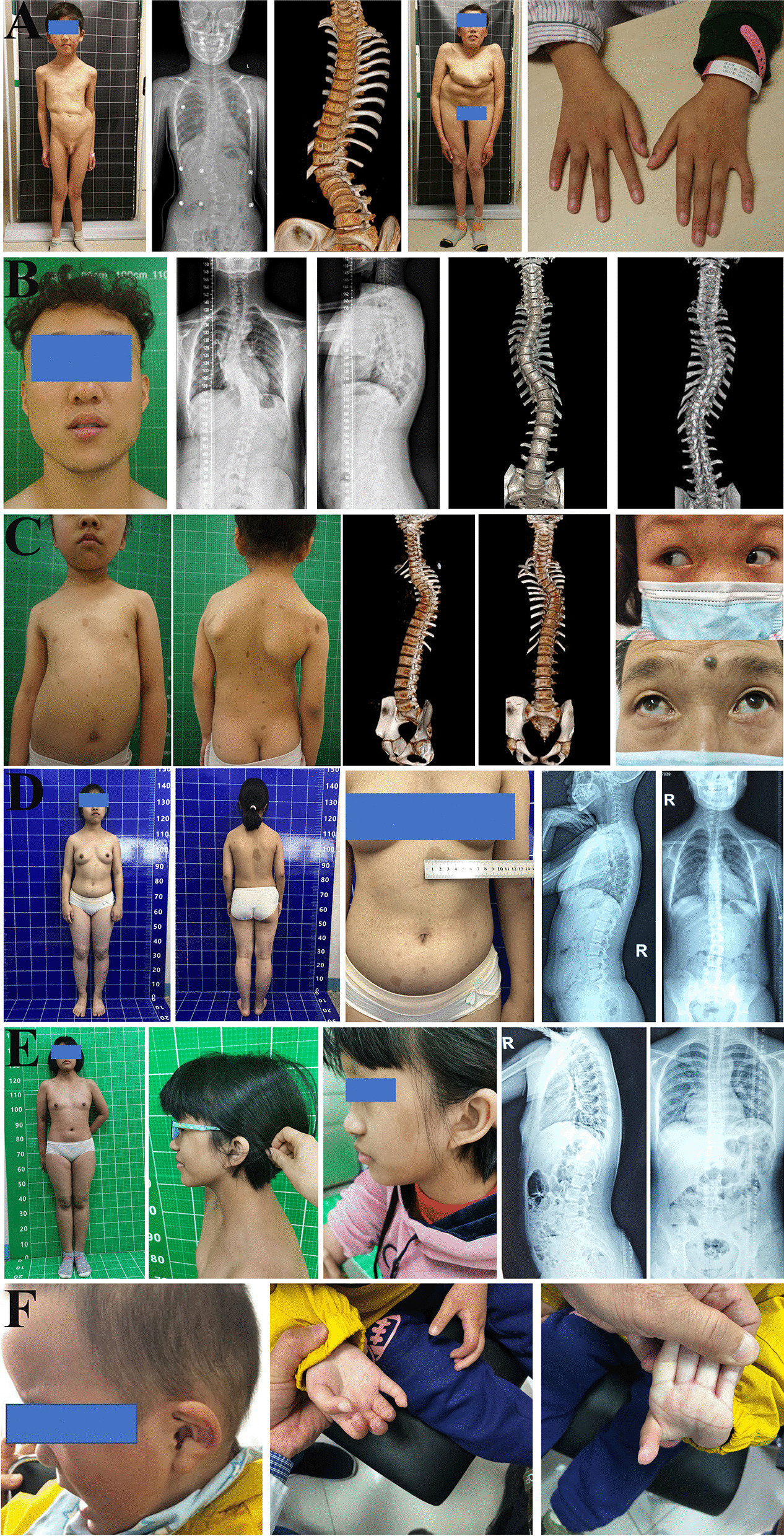
Representative clinical photographs of 6 patients with skeletal deformity. **a** Patient SCO2003P1972. **b** Patient SCO1908P0067. **c** Patient PCT2007P0019. **d** Patient SSS1910P0094. **e** Patient SSS2010P0110. **f** Patient RDD2001P0005

Patient SCO2003P1972 was a 7-year-old boy with early-onset scoliosis (Fig. 1a, Additional file 1: Fig. S1A, B). At 2 years old, he was diagnosed with congenital dislocation of hip joint (Table 1) and underwent a surgical reduction. A mild scoliosis was found during the hospitalization. At 7 years old, he was identified to have congenital scoliosis with segmentation failure of T10-L1 (Fig. 1a, Table 1). Physical examination showed ocular hypertelorism and intellectual disability (Fig. 1a, Table 1). ES revealed a pathogenic heterozygous nonsense variant c.2649G > A (p.Trp883Ter) in *FBN1* (Table 1), which is associated with Marfan syndrome (MIM:154700) [4]. Consistently, the proband presented Marfan syndrome-related phenotypes including mitral valve prolapse, mild arachnodactyly and scoliosis. This variant was inherited from his mother, who had severe scoliosis, arachnodactyly and dolichostenomelia (Fig. 1a). Through further analysis of the exome data, a de novo variant in *POGZ* c.1180_1181del (p.Met394ValfsTer9) (Table 1) was found in the proband. This variant was previously reported in patients with White–Sutton syndrome (WHSUS) (MIM:616364). WHSUS is characterized by intellectual disability, ocular abnormalities and brachydactyly [15], which largely overlapped with the phenotypes of this patient. Therefore, the complex phenotypes of this patient could be explained by a combined effect of variants in *POGZ* and *FBN1*.

#### Case 2

In case 2, the proband (SCO1908P0067) was referred to the clinic at the age of 15 years because of scoliosis (Fig. 1b, Table 1). He also presented pectus carinatum (Table 1). He experienced fracture at the age of 10 and 13. ES identified two pathogenic variants, including a de novo variant in *COL1A1* (c.1081C > T, p.Arg361Ter) and a maternally inherited variant in *FBN1* (c.1453C > T, p.Arg485Cys) (Table 1). These two reported variants led to dual molecular diagnosis consisting of osteogenesis imperfecta type I and Marfan syndrome [16, 17]. Although patients with osteogenesis imperfecta type I often present with short stature [18], the patient was tall (Height: 185 cm), which might be related with his second diagnosis of Marfan syndrome. Although the *FBN1* variant in our patient has been reported and considered as a pathogenic variant [17], no other features of Marfan Syndrome such as dolichostenomelia, arachnodactyly, joint laxity, velvety skin, ectopia lentis and cardiovascular manifestations were identified. This patient exemplified the agonistic effects of two Mendelian disorders on one trait (height in this case).

#### Case 3

Patient PCT2007P0019 was an 8-year-old girl. The proband had right tibia fracture at the age of 2 years. At around the age of 6 years, the patient developed scoliosis and razorback deformity, with her right shoulder slightly lower than left. The scoliosis progressed in the next 2 years (Additional file 1: Fig. S1D, E). A series of blended clinical phenotypes, such as blue sclera, cafe-au-Lait macules were observed as well (Fig. 1c, Table 1). Her father and younger brother also presented with blue sclera but did not have history of bone fracture. ES identified a pathogenic variant in *NF1* (c.2307del, p.Thr770LeufsTer21) and another pathogenic variant in *COL1A1* (c.2028 + 4A > G) (Table 1), both transmitted from her father. The *NF1* variant could lead to a frameshift of *NF1* (Table 1) and thus a loss-of-function effect, which is associated with neurofibromatosis type I and could explain the cafe-au-lait macules in this patient (Fig. 1c, Table 1). The *COL1A1* splicing variant (c.2028 + 4A > G) has been previously described to cause osteogenesis imperfecta [19], which could explain the recurrent bone fracture history in this patient. Interestingly, both neurofibromatosis type I and osteogenesis imperfecta could lead to scoliosis with incomplete penetrance [18, 20]. Therefore, the scoliotic phenotype in this patient might be caused by the synergistic effects of the *COL1A1* and the *NF1* variant.

#### Case 4

In our previous study, we reported a 6-year-old boy (SSS2008P0037) with short stature and craniofacial deformities, including depressed nasal bridge and long philtrum [21] (Table 1). Then the patient was detected to be double heterozygote for putatively pathogenic *ANKRD11* (c.2508dup, p.Leu837ThrfsTer81) and *COL11A1* (c.1180_1181del, p.Met394ValfsTer9) variants on ES (Table 1). The *ANKRD11* and *COL11A1* variants were confirmed as de novo and paternal status, respectively (Table 1). Clinical findings such as short stature and long philtrum were consistent with both KBG syndrome (MIM: 148050) and Marshall syndrome (MIM: 154780). In addition, the depressed nose in this patient is more likely to be associated with Marshall syndrome. The phenotypes of the patient together with the reported phenotypes of KBG syndrome and Marshall syndrome indicated overlapping clinical features in this case.

#### Case 5

This patient (SSS1910P0094) was an 8-year-old girl with idiopathic short stature and global developmental delay. She also presented widespread cafe-au-lait macules and mild lumbar scoliosis (Fig. 1d, Table 1). Additionally, her father (162 cm) and mother (140 cm) had short stature. Brain magnetic resonance (MR) showed abnormal signals in bilateral globus pallidus, thalamus, hippocampus and dentate nucleus of cerebellum were observed. Two pathogenic variants (Table 1), including a splicing variant in the *NF1* gene (c.6705-1G > A) and a frameshift variant in the *GLI2* gene (c.1189del, p.Val397CysfsTer124) were identified. Variants in *GLI2* have been shown to cause short stature, abnormal development of brain structures in Culler–Jones syndrome (MIM:615849) [22]. We suggested this patient’s presentation represents a mixture of distinct phenotypes, i.e., Cafe-au-Lait spots for NF type 1 (*NF1*, MIM:162200) and short stature for Culler–Jones syndrome (MIM:615849).

#### Case 6

Case 6 (SSS2010P0110) was a 12-year-old girl. She presented mild scoliosis, short stature, low posterior hairline, hyper pigmentation and webbed neck (Fig. 1e). ES identified a pathogenic nonsense variant in *TP63* (c.109C > T, p.Arg37Ter) and another pathogenic missense variant in *PTPN11* (c.1510A > G, p.Met504Val) (Table 1). Therefore, this patient reveived dual molecular diagnosis of Rapp–Hodgkin syndrome (MIM: 129400) and Noonan syndrome (MIM: 163950). However, this patient has minimal scoliosis and no major documented manifestation of Rapp-Hodgkin syndrome, suggesting a reduced expressivity of the *TP63* variant.

#### Case 7

A 3-year-old patient (RDD2001P0005) presented at birth with widespread interphalangeal joint contractures of the hands (Fig. 1f) and atrial septal defect (ASD) (Table 1). His father also had camptodactyly. ES revealed a paternally inherited heterozygous missense variant (c.3437A > G, p.Tyr1146Cys) in *FBN2* and a de novo heterozygous frameshift *ANKRD11* variant (c.3024_3025del, p.Lys1009GlyfsTer8) (Table 1). The *FBN2* variant causes Beals syndrome (Congenital contractual arachnodactyly) (MIM:121050), which is characterized by arachnodactyly and camptodactyly [23, 24]; The *ANKRD11* mutation causes KBG syndrome, which may contribute to the ASD in this patient [25]. Nevertheless, ASD has also been reported in patients with Beals syndrome [26], suggesting the overlapping phenotype associated with the dual molecular diagnosis.

#### Case 8

This case is a 5-year-old male (P19009402) with complex phenotypes. He presented congenital syndactyly of hand and foot (Additional file 1: Fig. S1F, G), cloverleaf skull, orbital hypertelorism, proptosis and midfacial hypoplasia (Table 1). He also had a history of malignant hyperthermia during general anesthesia. ES identified a de novo missense variant (c.755C > G, p.Ser252Trp) in *FGFR2* gene and a paternally inherited frameshift variant in *RYR1* gene (c.12788_12793dup, p.Glu4263_Gly4264dup) (Table 1). The *FGFR2* variant occurred in a known Apert syndrome hotspot [27]. Apert syndrome was characterized by craniosynostosis, proptosis, midfacial hypoplasia and severe syndactyly of the hands and feet [27], which are concordant with the phenotypes of this patient. Pathogenic *RYR1* variants are associated with malignant hyperthermia susceptibility 1(MHS 1) (MIM:145600) [28], which could explain the hyperthermia history in the patient.

## Discussion

The development of ES has significantly improved diagnostic yield of rare disease. Yang et al. found ES identified the underlying genetic defect in 25% of consecutive patients referred for evaluation of a possible genetic condition and 4.6% patients with blended phenotypes resulting from two single gene defects [10]. Farwell et al. found 11 (7.2%) in 152 probands with a positive or likely positive finding received a dual molecular diagnosis [29]. In a sizable cohort of 7374 patients, Posey et al. identified 2182 independent molecular diagnoses and identified two or more molecular diagnoses in 101 patients (4.9%) [9, 11]. In our study, we observed a relatively lower rate of dual molecular diagnosis in patients with skeletal diseases (≈ 1%), which might be attributed to the lower baseline molecular diagnosis rate as compared with those developmental disorder cohorts [30]. Most probands (5 out of 8) had family members with at least one of the diseases, consistent with the report by Balci et al. [31].

There are two kinds of effect caused by the interaction of two pathogenic variants. One was called “synergistic effect”, implicating that the combination of two mutational genes in patients would lead to more severe phenotypes. For instance, Xe et al. revealed a patient with variants in *CSNK2A1* and *TRPS1*, which resulted in a dual molecular diagnosis of tricho-rhino-phalangeal syndrome type I (TRPS I) and Okur-Chung neurodevelopmental syndrome (OCNDS). These two syndromes are both associated with short stature. Notably, this patient had a shorter stature as compared with other patients diagnosed with one of the diseases [12]. Moreover, Ye et al. reported a familial case with 13 patients affected by osteogenesis imperfecta (OI) type I, short stature and advanced bone age, with or without early-onset osteoarthritis and/or osteochondritis dissecans (SSOAOD). The proband was found to have two variants in *COL1A1* and *ACAN*. After comprehensive analysis of the height within the family, this study discovered a synergistic effect that the patients with two variants present severe short stature [32]. These manifestations resembled clinical presentations of one of our patients (SCO2003P1972). The kid was presented with a segmentation failure of T10-L1 of spinal vertebrae, which was an unusual phenotype of classical Marfan Syndrome. Additionally, a severe thoracolumbar curve (Cobb > 80°) were also observed in the kid. We concluded that the *POGZ* and *FBN1* variants both contributed to the skeletal deformity in this patient. However, in some patients who were diagnosed with dual molecular diagnosis, certain phenotypes may be opposite to that caused by a single genetic mutation. We proposed that these phenomena were caused by an antagonistic effect, as exemplified by the patient’s height in case 2. In addition, some of the cases with dual molecular diagnosis presented major phenotypic manifestation of one genetic disorder and only minor phenotypes of the other, as exemplified by our case 6 and case 8. This suggests that the genetic terminologies ‘variable expressivity’ and ‘incomplete penetrance’ are also applicable to the condition of dual molecular diagnosis.

All the dual diagnosis conditions in our study were identified through ES. However, the high expense of ES hinders it from being either a stand-alone or a first-tier diagnostic approach, especially in developing country. Therefore, selecting the most appropriate molecular diagnostic tool is important when ordering genetic testing. Single-gene testing should be recommended when the clinical features for a patient were typical for a specific disorder and the association between the disorder and a single gene was well-established [33]. For example, *TBX6*-associated congenital scoliosis was characterized by simple hemi‐/wedge‐shaped vertebrae in the lower half of the spine [34–36]. Furthermore, our previous study found that a novel de novo* FBN1* variant could explain the Marfanoid–progeroid–lipodystrophy syndrome (MIM:616914) [37]. Under these circumstances, single-gene testing should be ordered. In contrast, in cases with complex phenotypes that cannot be explained by one genetic defect as shown in our examples, ES can be utilized as first line test which could shorten the diagnostic odyssey of the patients [33].

In conclusion, this study revealed the molecular diagnoses and complex diagnostic odyssey of dual molecular diagnosis through analyzing the clinical traits and genetic data of bone deformity in eight patients.

## Conclusions

This study revealed the complicated skeletal phenotypes associated with dual molecular diagnosis. Exome sequencing represents a powerful tool to detect these complex conditions.

## Methods

### Study design

This is a retrospective study which reports the clinical and genetic characteristics of a group of patients with dual molecular diagnosis.

### Subjects

Cases with skeletal disorders from the DISCO study (http://www.discostudy.org/) who underwent ES were included. The chief complains include early-onset scoliosis (EOS) (N = 447), short stature (N = 561), and congenital limb malformations (CLM) (N = 100). Deep phenotyping and radiological examinations including X-ray, computed tomography (CT), and magnetic resonance imaging (MRI) were performed on each patient as previously reported [4, 38], (Fan et al., *Journal of Genetics and Genomics*, 2021, in press). Written informed consent was obtained from every participant; if the participant was younger than 16 years old, written informed consent was obtained from their parents or legal guardians. The study was approved by the institutional review board of PUMCH (JS-2364), Beijing Jishuitan Hospital (201808-09) and the Second Affiliated Hospital of Guangxi Medical University (G-1-1).

### Exome sequencing and variant interpretation

ES was performed on DNA extracted from blood of all 8 probands and their family members. The sequencing data were analyzed and annotated using an in-house developed analytical pipeline, Peking Union Medical college hospital Pipeline (PUMP) [39–41]. All variants were presumed to be pathogenic were subjected to Sanger sequencing.

### Identification of dual molecular diagnosis

Patients with more than one molecular diagnosis from the included patients were selected for analyses. Each molecular diagnosis was manually curated based on the pathogenicity of the variants and the Mendelian expectations for inheritance mode. The pathogenicity of the variants was evaluated according to the American College of Medical Genetics and Genomics (ACMG) guidelines [42]. The Mendelian expectations for inheritance mode include autosomal dominant (AD) inheritance, autosomal recessive (AR) inheritance and X-linked dominant/recessive (XLD/XLR) inheritance. For AD/XLD traits, one heterozygous pathogenic/likely pathogenic variant is sufficient to establish a molecular diagnosis. For AR/XLR traits, one homozygous, one hemizygous or one pair of compound heterozygous pathogenic/likely pathogenic variants are required for a molecular diagnosis.

## Supplementary Information

**Additional file 1:** Supplementary clinical photographs of patients in our study.

## Data Availability

The datasets used and/or analyzed during the current study are available from the corresponding author on reasonable request.

## Abbreviations

*COL1A1*: Collagen, type I, alpha-1
*NF1*: Neurofibromatosis, type I
*FBN1*: Fibrilin-1
*FBN2*: Fibrillin-2
*PTPN11*: Protein tyrosine phosphatase, non-receptor type 11 gene
ES: Exome sequencing
EOS: Early-onset scoliosis
*WHSUS*: White–Sutton syndrome
*Gli2*: GLI-Kruppel family member 2
*ANKRD11*: Ankyrin repeat domain-containing protein 11
*FGFR2*: Fibroblast growth factor receptor 2
*RYR1*: Ryanodine receptor 1
*CSNK2A1*: Casein kinase II, alpha subunit
*TRPS1*: Trichorhinophalangeal syndrome type I
