# Supplementary figures and images for: Delineation of dual molecular diagnosis in patients with skeletal deformity
Source: Orphanet J Rare Dis. 2022 Mar 28;17:139. doi: 10.1186/s13023-022-02293-x (PMC8962553; doi:10.1186/s13023-022-02293-x)

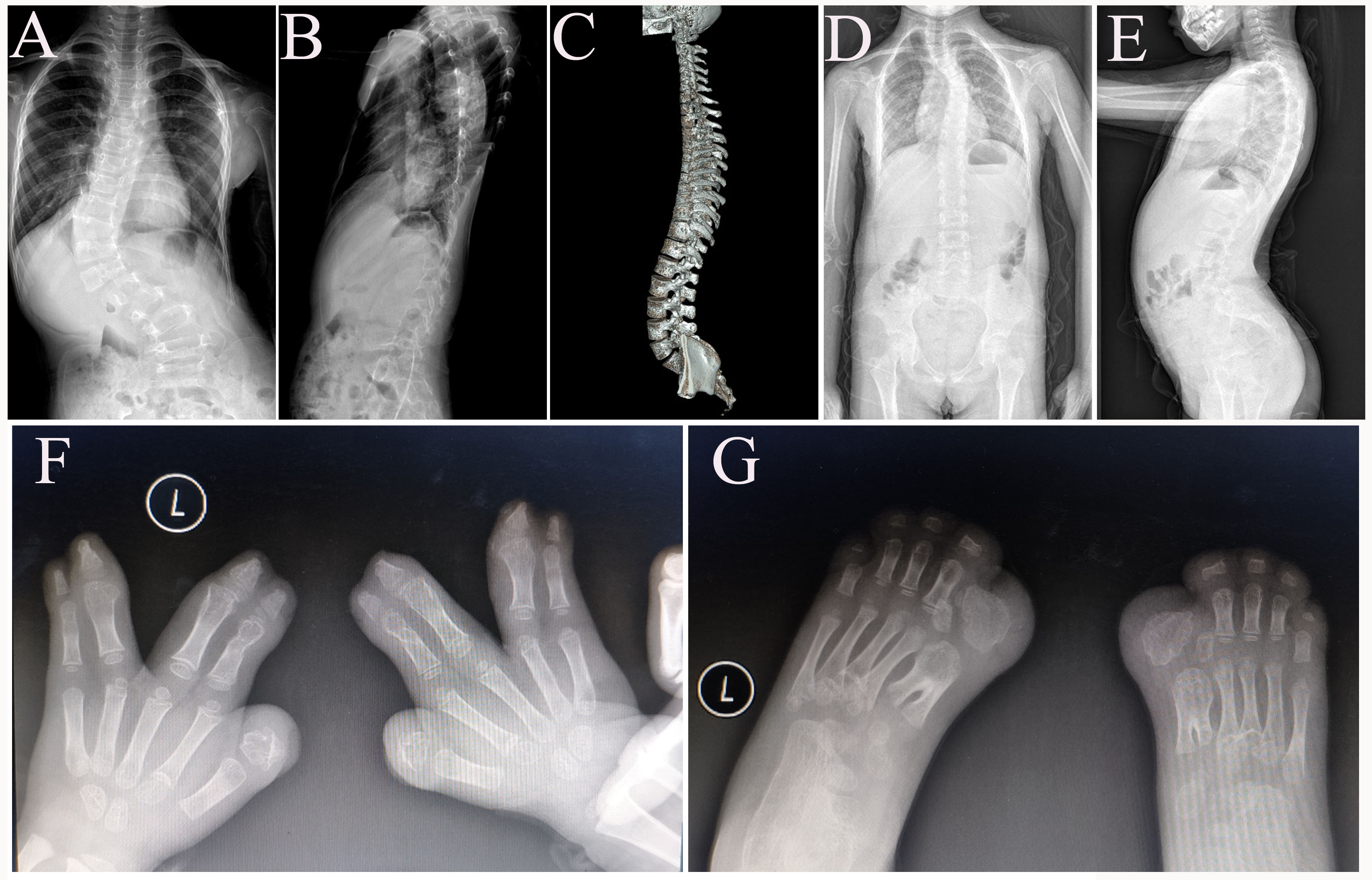

Supplement: Supplementary file 1 — Additional file 1: Supplementary clinical photographs of patients in our study. [file 13023_2022_2293_MOESM1_ESM.jpg]
